# Supplementary material for: Hormonal steroids induce multidrug resistance and stress response genes in Neisseria gonorrhoeae by binding to MtrR
Source: Nat Commun. 2024 Feb 7;15:1153. doi: 10.1038/s41467-024-45195-1 (PMC10850145; doi:10.1038/s41467-024-45195-1)
Supplement: Supplementary file 1 — Supplementary Information [file 41467_2024_45195_MOESM1_ESM.pdf]

Supplementary tables

Table S1. Steroidal hormones are substrates of the MtrCDE efflux pump

	MIC (μg/mL)		
	Progesterone	β-estradiol	Testosterone
FA19	40 (127 μM)	>320 (1175 μM)	160 (555 μM)
FA19 *mtrD*::kan (KH14)	20 (64 μM)	320 (1175 μM)	40 (139 μM)

*Minimal inhibition concentration is defined by the lowest concentration at which visible growth is prevented.

Table S2. Strains and plasmids

Strains	Description	Reference
FA19	Cervical isolate from a woman with disseminated gonococcal infection. Possesses a WT *mtr* locus	[1]
JF1	FA19 Δ*mtrR*	[2]
FA19::P*lptA*-*lacZ*	FA19 carrying pLES94-*lptA*	[3]
JC106	FA19 carrying pLES94-*mtrC*	This study
JC107	JF1 carrying pLES94-*mtrC*	This study
JC108	FA19 carrying pLES94-*rpoH*	This study
JC109	JF1 carrying pLES94-*rpoH*	This study
JC110	FA19 carrying pLES94-*ngo1249*	This study
JC89	JF1 carrying pLES94-*mtrC* and pGCC3-*mtrR*	This study
JC90	JF1 carrying pLES94-*mtrC* and pGCC3-*mtrR*-W136L	This study
JC91	JF1 carrying pLES94-*rpoH* and pGCC3-*mtrR*	This study
JC92	JF1 carrying pLES94-*rpoH* and pGCC3-*mtrR*-W136L	This study
JC93	JF1 carrying pLES94-*mtrC* and pGCC3-*mtrR*-R176E	This study
JC94	JF1 carrying pLES94-*rpoH* and pGCC3-*mtrR*-R176E	This study
JC101	JF1 carrying pLES94-*mtrC* and pGCC3-*mtrR*-D171A	This study
JC102	JF1 carrying pLES94-*rpoH* and pGCC3-*mtrR*-D171A	This study
**Plasmid**	**Description**	**Reference**
pLES94	Vector carrying a promoterless *lacZ* and *proAB* homology regions for recombination in *N. gonorrhoeae*	[4]
pLES94-*mtrC*	pLES94 with a *mtrC-lacZ* transcriptional and translational fusion	This study
pLES94-*rpoH*	pLES94 with a *rpoH-lacZ* transcriptional and translational fusion	This study
pLES94-*lptA*	pLES94 with a *lptA-lacZ* transcriptional and translational fusion	[3]
pLES94-*ngo1249*	pLES94 with a *ngo1249-lacZ* transcriptional and translational fusion	This study
pGCC3	Vector for genetic complementation in gonococci. Carries the *lctP-aspC* loci for homologous recombination. Erm^R, Km^R	[5]
pGCC3-*mtrR*	pGCC3 containing a copy of the *mtrR* locus encoding MtrR from its own promoter	This study
pGCC3-*mtrR*-W136L	pGCC3 containing a copy of the *mtrR* locus encoding mutant MtrR (W136L) from its own promoter	This study
pGCC3-*mtrR*-R176E	pGCC3 containing a copy of the *mtrR* locus encoding mutant MtrR (R176E) from its own promoter	This study
pGCC3-*mtrR*-D171A	pGCC3 containing a copy of the *mtrR* locus encoding mutant MtrR (D171A) from its own promoter	This study
mtrR-pMCSG7	pMCSG7 bearing the WT *mtrR* CDS	[6]
pGAB020RGB	Point mutation derivative of *mtrR*-pMCSG7 obtained with DpnI-mediated site-directed mutagenesis encoding *mtrR* W136L	[6]
pGAB018RGB	Point mutation derivative of *mtrR*-pMCSG7 obtained with DpnI-mediated site-directed mutagenesis encoding *mtrR* R176E	[6]

pGMH012RGB	Point mutation derivative of *mtrR*-pMCSG7 obtained with DpnI-mediated site-directed mutagenesis encoding *mtrR* D171A	This study
pGMH013RGB	Point mutation derivative of *mtrR*-pMCSG7 obtained with DpnI-mediated site-directed mutagenesis encoding *mtrR* W136A	This Study
pGMH014RGB	Point mutation derivative of *mtrR*-pMCSG7 obtained with DpnI-mediated site-directed mutagenesis encoding *mtrR* Q133A	This Study

Table S3 Oligonucleotides used in this study

Oligonucleotide	Sequence (5'-3')
mtrC-lacZ-F	TTTGGATCCGGTTTGACGAGGGC
mtrC-lacZ-R	AGGGATCCGAAGCATAAAAAGCCATTATTT
rpoH-lacZ-F	GGGATCCGGCGAAACGCCCTATATGAA
rpoH-lacZ-R	GGGATCCCGATTCATTTGGGGCATTTCCTTT
1249-lacZ-F	CCGGATCCAAATCTTTTTGAACCATA
1249-lacZ-R	ATGGATCCTTAAACATTTTCTTTTCCTTTC
proABFw	AACTCGATGGAAGTGCTGCTGGT
lacZRv	AACTGTTGGGAAGGGCGATCGGT
pacImtrR-Fw	GGTTAATTAACGCCTTAGAAGCATAAAAAGCCAT
pmeImtrR-Rv	GGGTTTAAACTTATTTCCGGCGCAGGCAG
pGCC3Rv	AACCCTTAATATAACTTCGTATAATG
mtrR_qRT_R	GTGGATGTCGTTGCTTTGCA
lctPqFw	CGCCATCAAACTTTTCTACTTCGG
mtrR-midRv	AAGCATCAGGTGTTCTTTGGTTTT
mtrR-midFw	ATGAGAAAAACCAAAACCGAAGCC

Supplementary figures and legends

Figure S1:

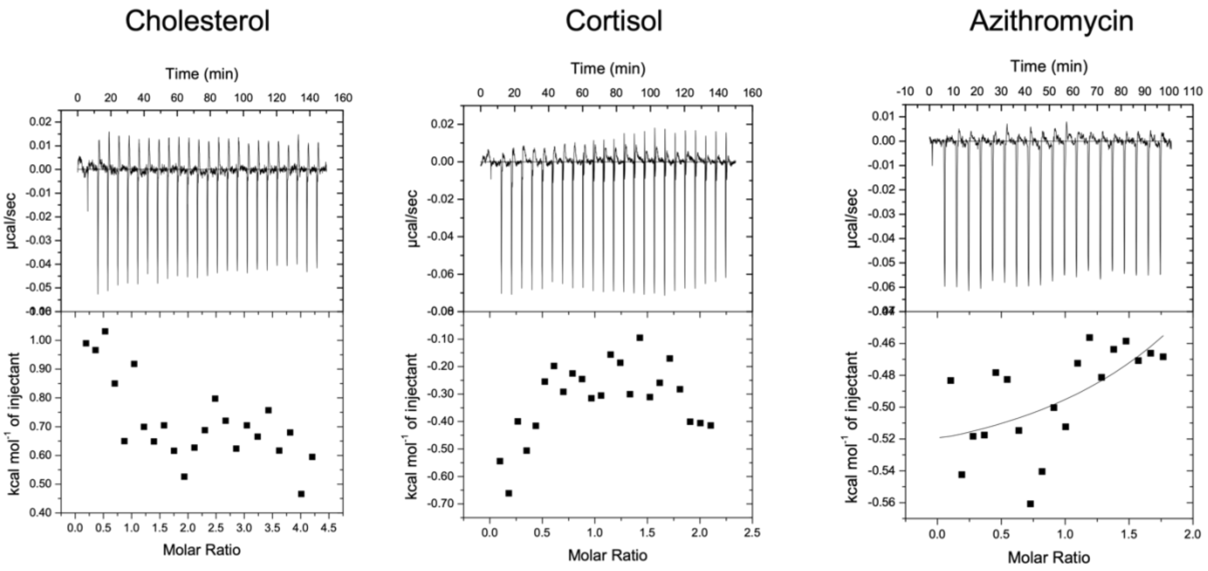

Figure S1: Characterization of the MtrR ligand binding pocket:
Isothermal titration calorimetry thermograms and resulting binding isotherms for binding reactions between MtrR WT with cholesterol, cortisol, and azithromycin. Purified MtrR was concentrated to 20 µM in purification buffer plus 0.6 - 1% MeOH. Titrations with 20 µM MtrR in the sample cell and 100 µM (cholesterol), 200 µM (cortisol), or 250 µM (azithromycin) in the syringe were performed using a VP-ITC microcalorimeter (Microcal Inc.). ITC experiments were conducted at 25 °C with a stirring speed of 199 rpm. Data were analyzed using ORIGIN 7.0. At least three experimental measurements, technical and some biological replicates, were averaged for each reported binding constant.

Figure S2.

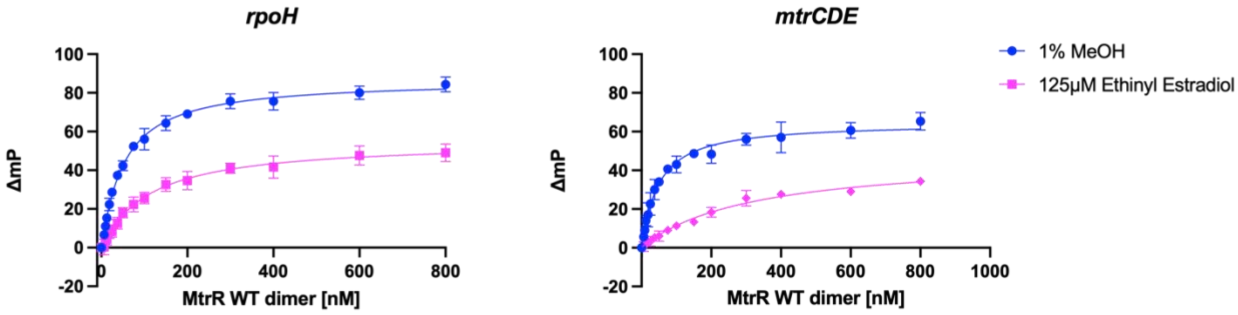

	rpoH 27mer		*mtrCDE* 21mer	
	K_d [nM]	Fold increase in K_d	K_d [nM]	Fold increase in K_d
1% MeOH	46.3 ± 6.3	---	43.04 ± 11.6	---
Ethinyl Estradiol (125 μM)	102.5 ± 29.2	2.2	313.3 ± 97.9	7.3

Figure S2: Presence of Ethinyl Estradiol influences binding of MtrR to *rpoH* and *mtrCDE* operators. Plate based Fluorescence polarization DNA-binding isotherms reveal decreases in the binding affinity of MtrR for the *rpoH* and *mtrCDE* operators in the presence of ethinyl estradiol. Experiments were run on the three independent experimental measurements were averaged for each reported binding constant. Data are represented by the mean values (point) +/- SEM (error bar).

Figure S3

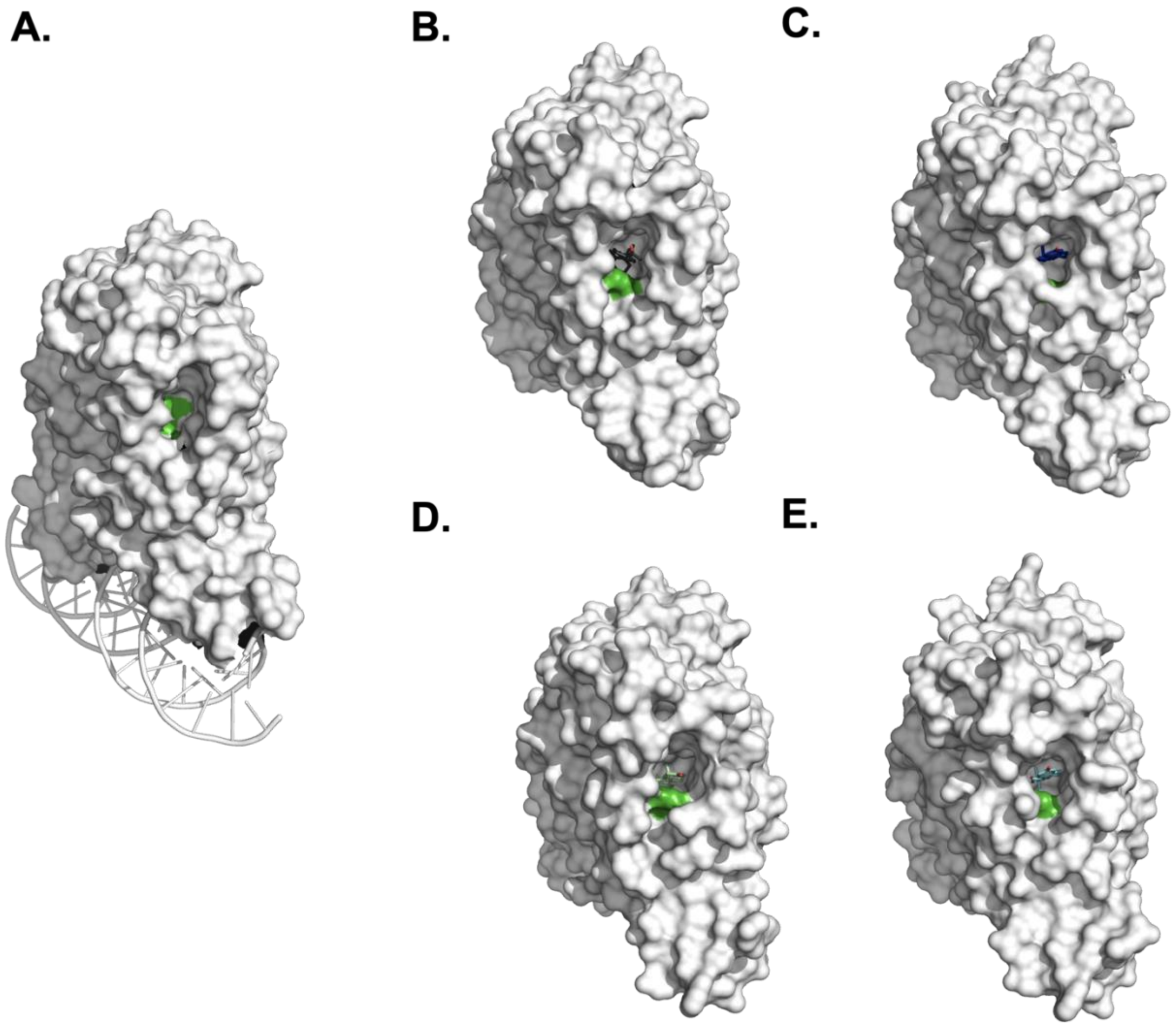

Figure S3: Entrance to ligand binding pocket. Surface view of MtrR bound to (**A**) the *mtrCDE* operator (PDB: 7JU3) [7], (**B**) progesterone colored charcoal, (**C**) β-estradiol blue, (**D**) testosterone pale green, and (**E)** ethinyl estrogen cyan. W136 painted in green.

Figure S4

A.

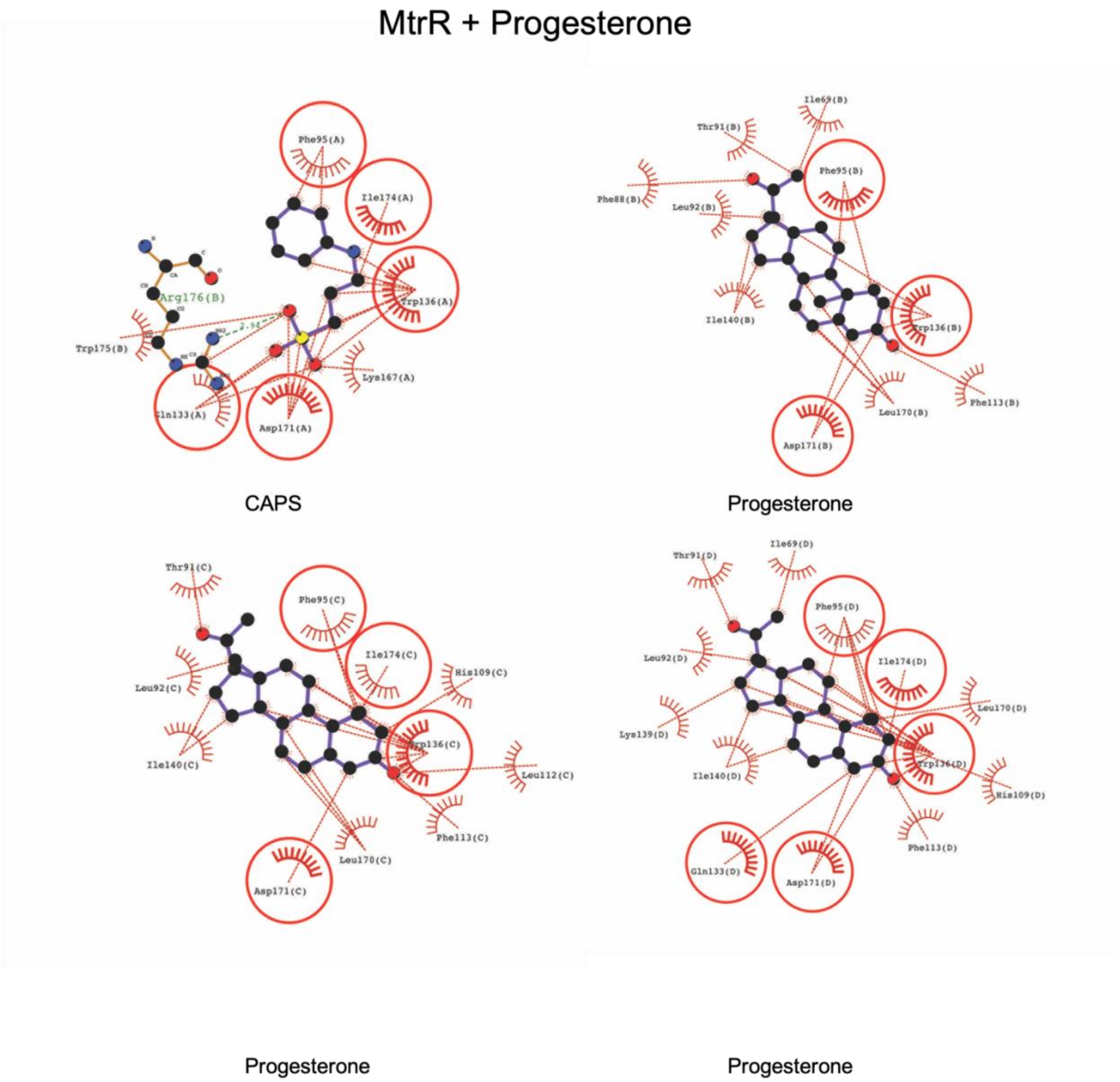
MtrR + Progesterone
Phe95(A)
Ile174(A)
Arg176(B)
Trp136(A)
Trp175(B)
Lys167(A)
Gln133(A)
Asp171(A)
CAPS
Ile69(B)
Thr91(B)
Phe95(B)
Phe88(B)
Leu92(B)
Ile140(B)
Trp136(B)
Phe113(B)
Leu170(B)
Asp171(B)
Progesterone
Thr91(C)
Phe95(C)
Ile174(C)
His109(C)
Leu92(C)
Trp136(C)
Leu112(C)
Ile140(C)
Phe113(C)
Leu170(C)
Asp171(C)
Ile69(D)
Thr91(D)
Phe95(D)
Leu92(D)
Ile174(D)
Leu170(D)
Lys139(D)
Trp136(D)
Ile140(D)
His109(D)
Phe113(D)
Gln133(D)
Asp171(D)
Progesterone
Progesterone

B.

MtrR + Testosterone

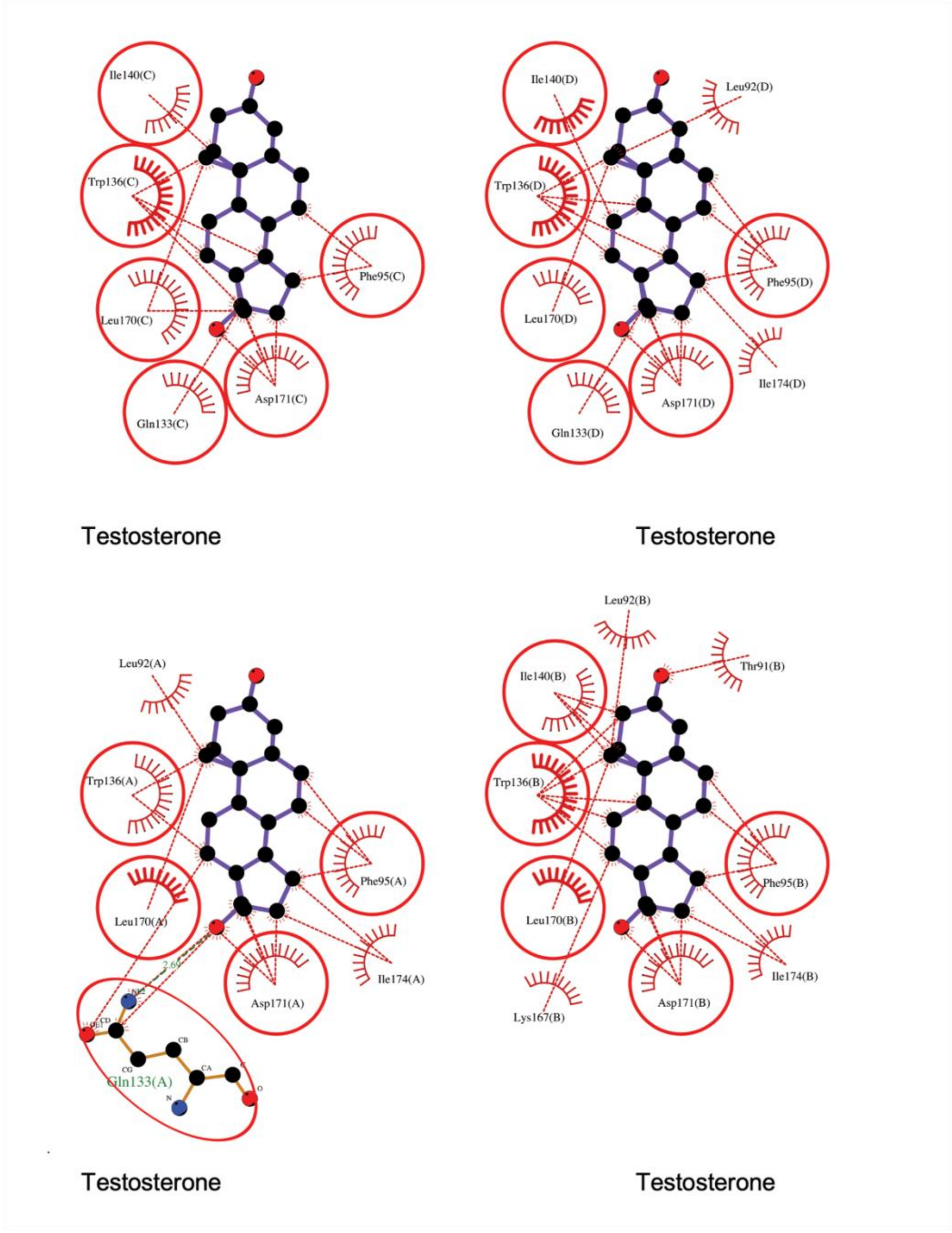

C.

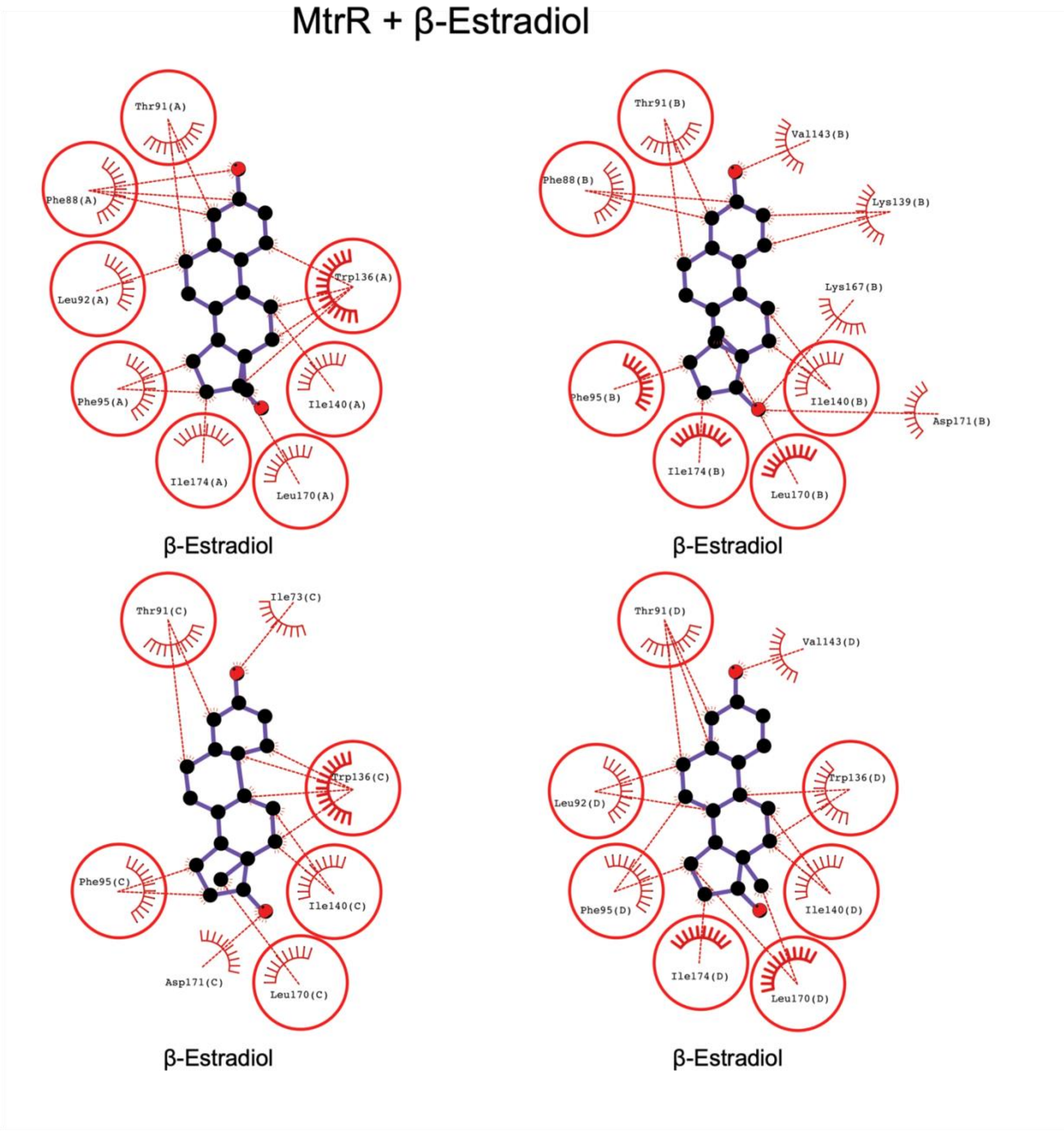
MtrR + β-Estradiol
Thr91(A)
Phe88(A)
Leu92(A)
Trp136(A)
Phe95(A)
Ile140(A)
Ile174(A)
Leu170(A)
β-Estradiol
Thr91(B)
Val143(B)
Phe88(B)
Lys139(B)
Lys167(B)
Phe95(B)
Ile140(B)
Asp171(B)
Ile174(B)
Leu170(B)
β-Estradiol
Thr91(C)
Ile73(C)
Trp136(C)
Phe95(C)
Ile140(C)
Asp171(C)
Leu170(C)
β-Estradiol
Thr91(D)
Val143(D)
Leu92(D)
Trp136(D)
Phe95(D)
Ile140(D)
Ile174(D)
Leu170(D)
β-Estradiol

D.

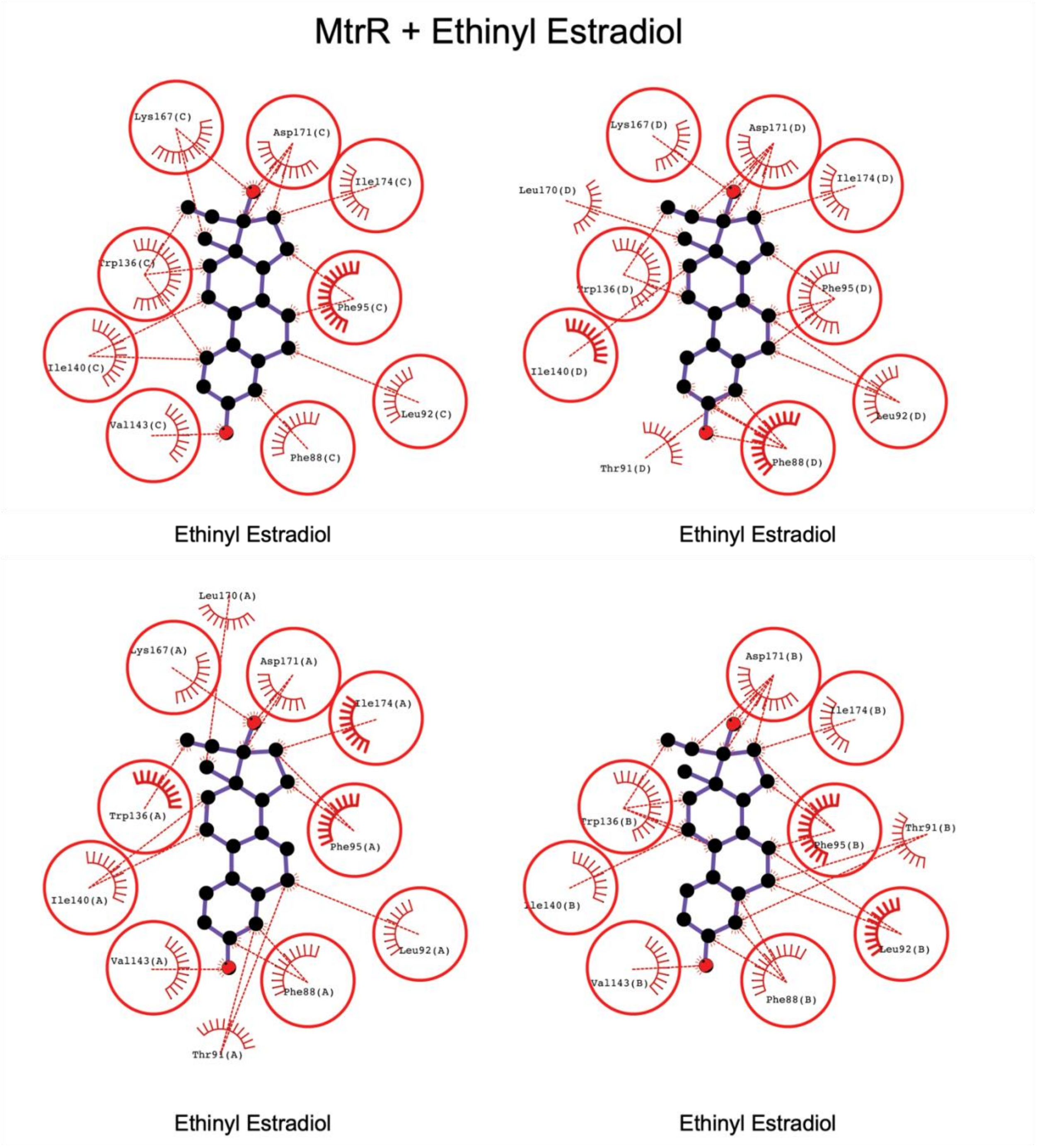

Figure S4: Interaction of MtrR and steroids.
LigPlot+ v.2.2 was used to highlight residues important for MtrR and ligand binding. A-D. Interactions between MtrR-PTR, MtrR-TES, MtrR-EST, and MtrR-NDR, respectively, in each binding pocket in the ASU (4 subunits per ASU). The chemical structure of each ligand is shown.

Potential hydrogen bonds are shown when the maximum hydrogen bond donor-acceptor distances are between 2.70 – 3.35 Å. Potential non-bonded contacts, i.e., Van der Waals/hydrophobic interactions, between 2.90 – 3.90 Å are also shown. MtrR residues involved in hydrophobic contacts are shown with red eyelashes with ticked lines to the corresponding atoms involved. Hydrogen bonds and their lengths in angstrom are shown in green. Residues with equivalent interactions in all ASU's are circled in red.

Figure S5

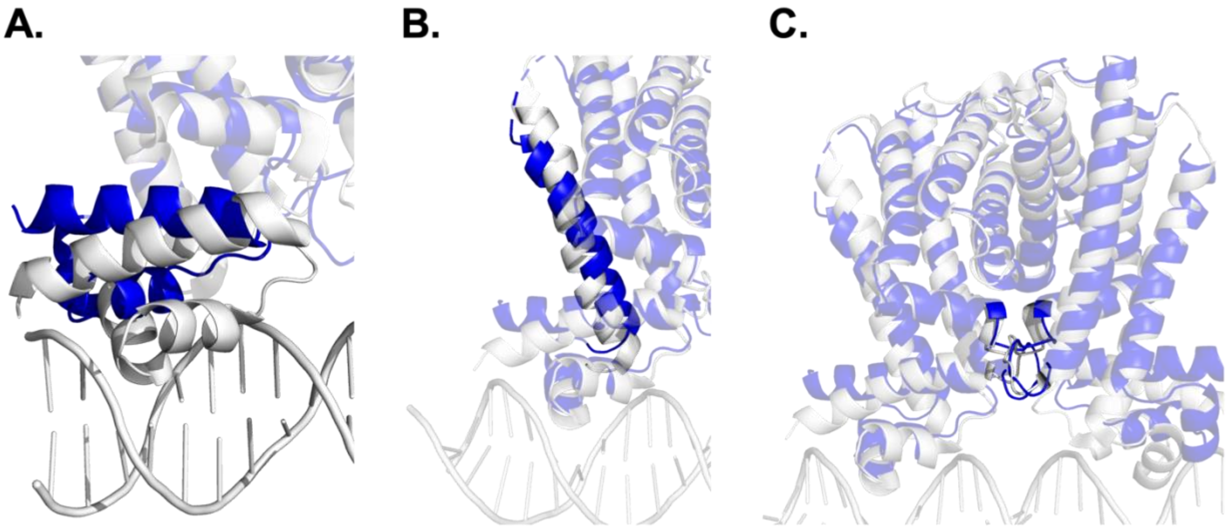

Figure S5: Induction movements of MtrR. Overlay of induced MtrR bound to β-estradiol (blue) and MtrR bound to the *mtrCDE* operator site (white). Key conformational changes occur in the HTH domain (**A**), α4 (**B**), and the loop formed by residues 114-122, between helices α6 and α7 (**C**).

Figure S6

A

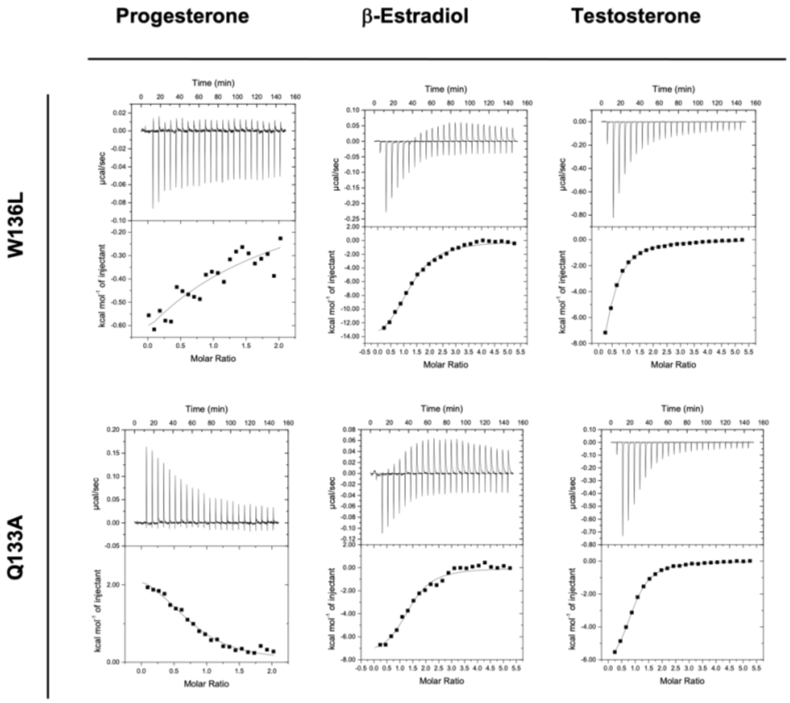

MtrR	Progesterone (K_d)	β-Estradiol (K_d)	Testosterone (K_d)
WT	2.75 ± 0.7 μM	1.67 ± 1.1 μM	3.53 ± 2.9 μM
W136L	89.8 ± 39.3 μM	0.87 ± 0.47 μM	9.42 ± 1.90 μM
Q133A	3.45 ± 1.1 μM	1.14 ± 0.2 μM	3.35 ± 0.1 μM

B

MtrR D171A + Ethinyl Estradiol

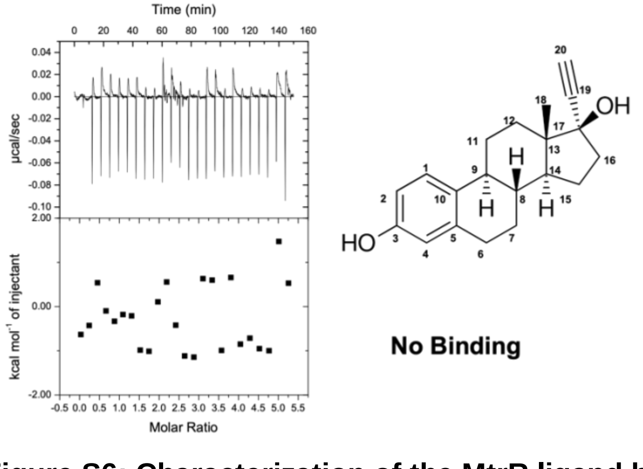

Figure S6: Characterization of the MtrR ligand binding pocket:
Isothermal titration calorimetry thermograms and resulting binding isotherms for binding reactions between **(A)** MtrR W136L and Q133A with progesterone, β-estradiol, or testosterone and **(B)** MtrR

D171A with ethinyl estradiol. Purified MtrR mutants were concentrated to 20 µM in purification buffer plus 1% MeOH. Titrations with 20 or 5 µM MtrR in the sample cell and 125, 200, or 500 µM compound in the syringe were performed using a VP-ITC microcalorimeter (Microcal Inc.). ITC experiments were conducted at 25 °C with a stirring speed of 199 rpm. Data were analyzed using ORIGIN 7.0. At least three experimental measurements, technical and some biological replicates, were averaged for each reported binding constant.

Figure S7

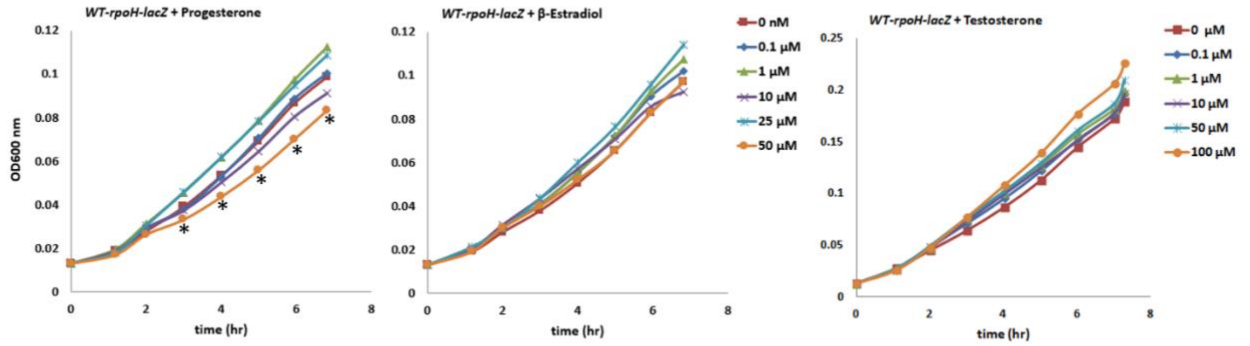

Fig S7. Growth curve of strain JC108 in increasing concentrations of hormones. 10^8 CFU/mL of gonococci were grown statically at 37 ºC, 5% (v/v) CO_2 in 100 µL of GC-broth in 96-well polystyrene plates. * Statistical differences between 1 and 50 µM hormone using a one-tailed t-test; in all cases the p value was < 0.05.

Figure S8

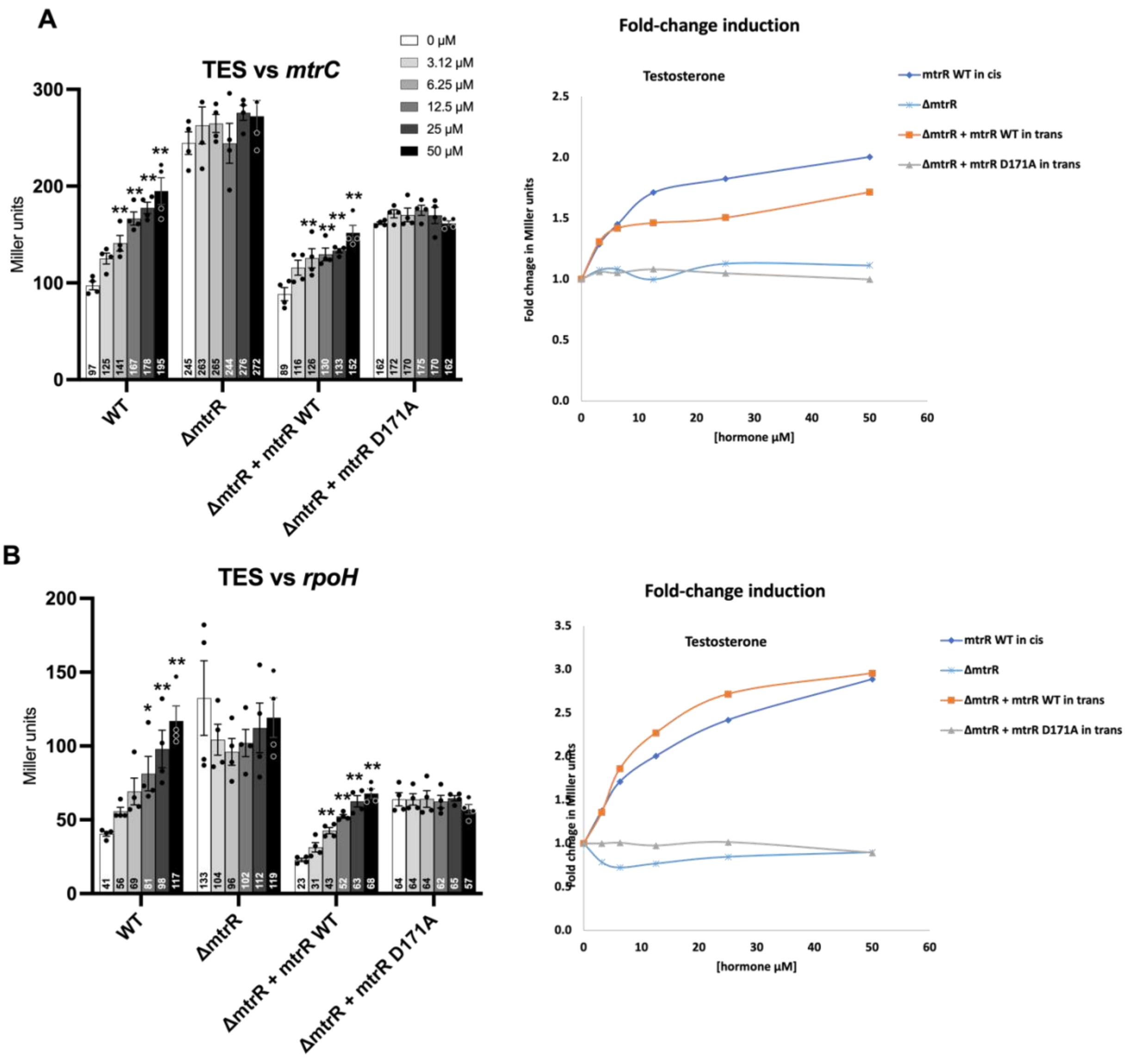

Fig S8. Testosterone induces the expression of gonococcal genes *mtrC* and *rpoH* in an MtrR-dependent manner. 10^8 CFU/mL of wild-type (WT) *mtrR* bearing strains (JC106 and JC108), *mtrR* deletion mutants (JC107 and JC109) and complemented strains in trans with WT *mtrR* (JC89 and JC91) and mutant allele *mtrR* D171A (JC101 and JC102) were grown in GC-broth to late exponential phase in increasing concentrations of testosterone, statically in 96-well plates (37 ^oC, 5% v/v CO_2). Expression of *mtrC* (A) and *rpoH* (B) was measured from the transcriptional and translational *lacZ* fusions and expressed as a corresponding β-galactosidase activity in Miller units as described in Methods. Data are represented by the mean (bar) + SEM (error bar) of n=4 biological samples in the bar graphs and as fold change in Miller units relative

to the zero-hormone control in the line graphs. The experiments were performed at least thrice with reproducible results. Statistics: ANOVA test and a Dunnett's Multiple Comparison post-test (*, ** statistically different from 0 µM at $p<0.05$ and 0.01)

Figure S9

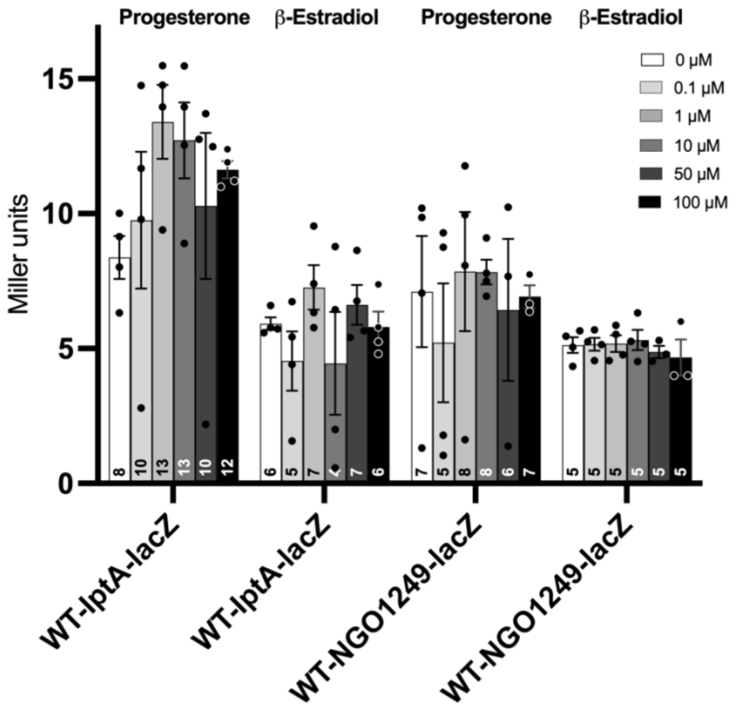

Fig S9. Steroidal hormones do not affect the expression of *lptA* and *ngo1249,* genes outside the MtrR regulon. Steroidal hormones did not affect the expression of two genes not regulated by MtrR, which validates that the differences we observed in expression levels for *mtrC* and *rpoH* in the presence of the hormones are due to MtrR regulation and not to a polar effect of the hormones at the locus where these recombinant genetic constructions were placed. 10^8 CFU/mL of gonococci were grown in GC-broth to late exponential phase in increasing concentrations of progesterone or β-estradiol, statically in 96-well plates (37 ^{o}C, 5% v/v CO_2). Expression of *lptA* (in strain FA19::PlptA-lacZ) and *ngo1249* (in strain JC110) was measured using transcriptional and translational *lacZ* fusions and the corresponding β-galactosidase activities were expressed in Miller units. Data are represented by the mean (bar) + SEM (error bar) of n=4 biological samples. The experiments were performed at least twice with reproducible results. There were not statistical differences by addition of the hormones by performing an ANOVA test and a Dunnett's Multiple Comparison post-test.

Figure S10

A

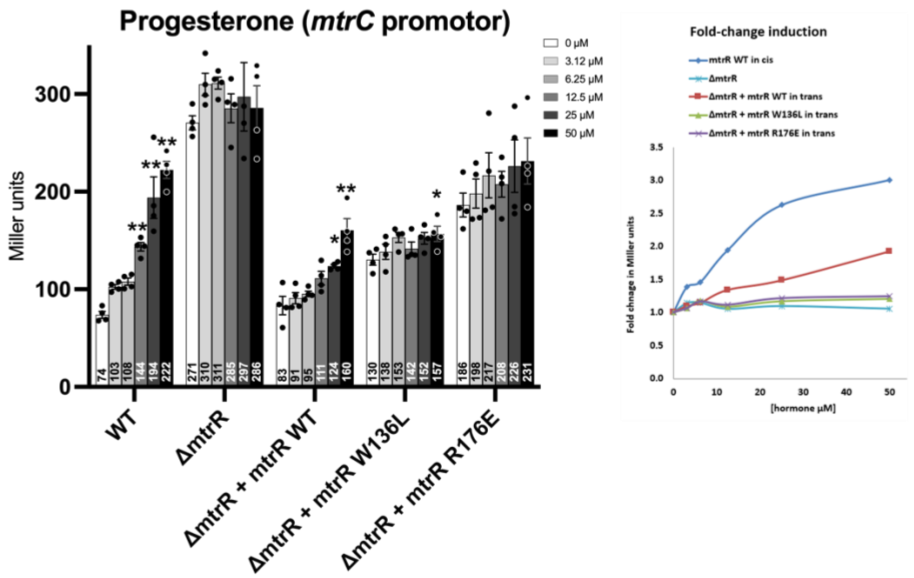

B

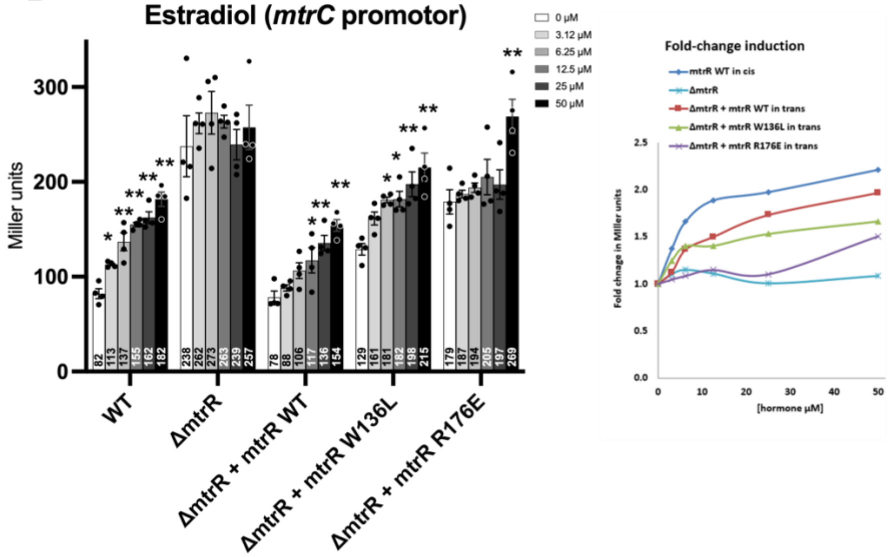

C

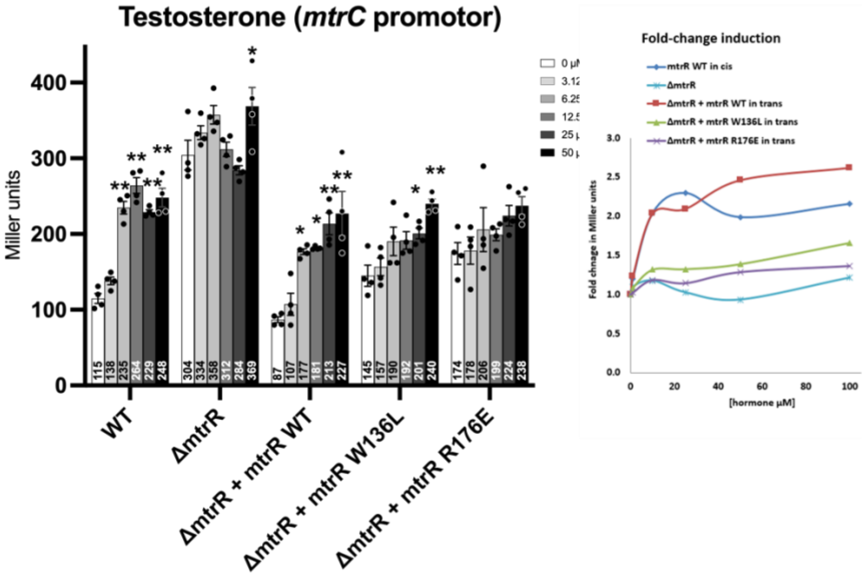
Testosterone (mtrC promotor)
Miller units
400
300
200
100
0
WT
ΔmtrR
ΔmtrR + mtrR WT
ΔmtrR + mtrR W136L
ΔmtrR + mtrR R176E
Fold-change induction
mtrR WT in cis
ΔmtrR
ΔmtrR + mtrR WT in trans
ΔmtrR + mtrR W136L in trans
ΔmtrR + mtrR R176E in trans
Fold change in Miller units
[hormone µM]

D

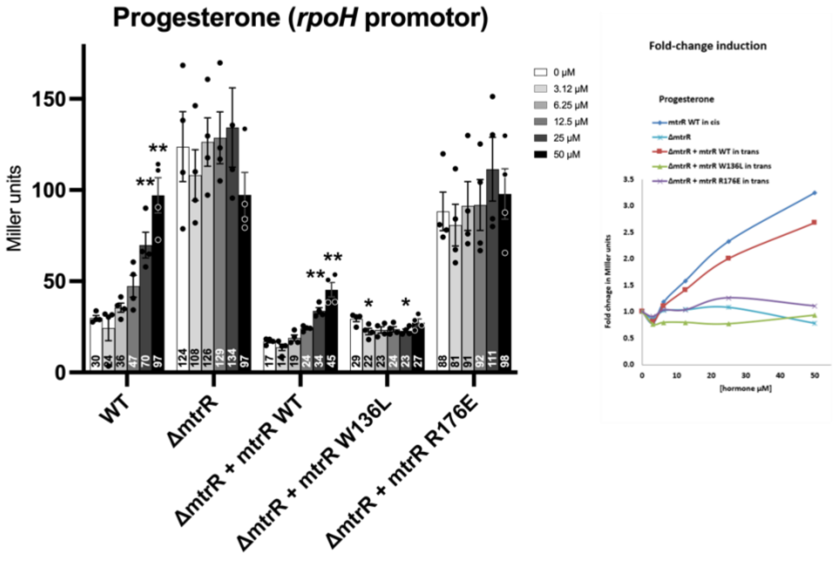
Progesterone (rpoH promotor)
Miller units
150
100
50
0
0 µM
3.12 µM
6.25 µM
12.5 µM
25 µM
50 µM
WT
ΔmtrR
ΔmtrR + mtrR WT
ΔmtrR + mtrR W136L
ΔmtrR + mtrR R176E
Fold-change induction
Progesterone
mtrR WT in cis
ΔmtrR
ΔmtrR + mtrR WT in trans
ΔmtrR + mtrR W136L in trans
ΔmtrR + mtrR R176E in trans
[hormone µM]

E

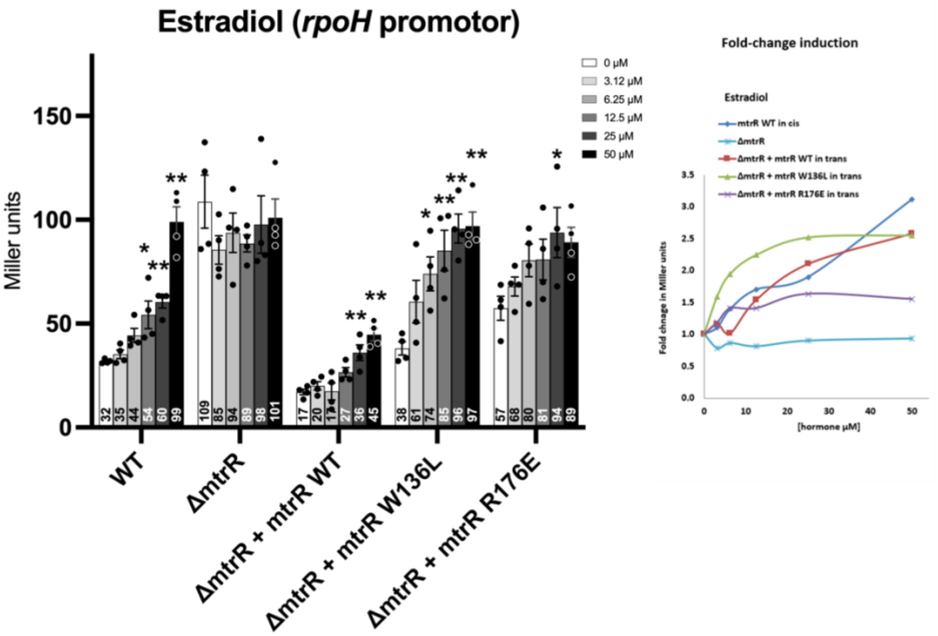

F

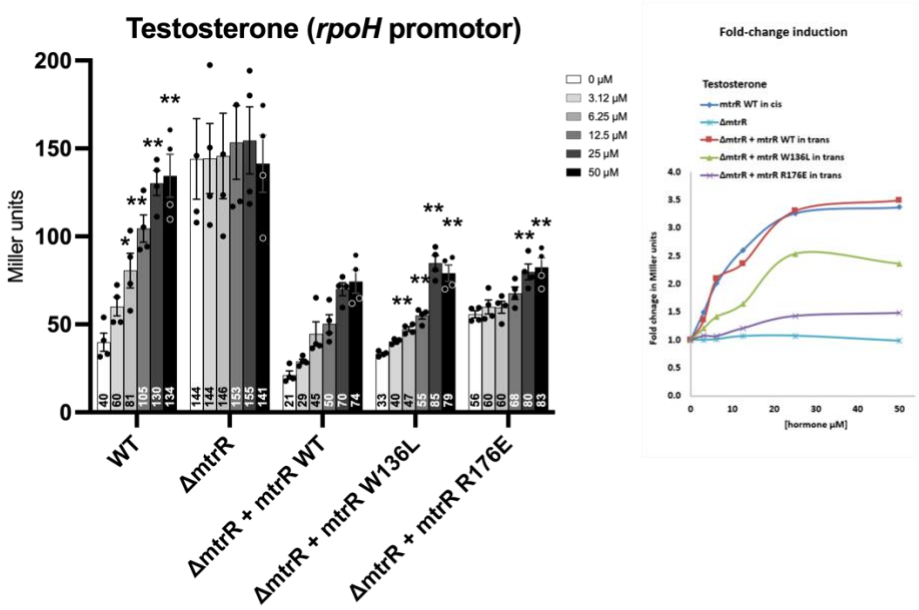

Fig S10. Steroidal hormones induce the expression of gonococcal genes *mtrC* and *rpoH* in an MtrR-dependent manner. 10^8 CFU/mL of wild-type (WT) *mtrR* bearing strains (JC106 and JC108), *mtrR* deletion mutants (JC107 and JC109) and complemented strains in trans with WT *mtrR* (JC89 and JC91) and mutant alleles *mtrR* W136L (JC90 and JC92) and *mtrR* R176E (JC93 and JC94) were grown in GC-broth to late exponential phase in increasing concentrations of progesterone (A and D), estradiol (B and E) and testosterone (C and F), statically in 96-well plates

(37 ^{o}C, 5% v/v CO_2). Expression of *mtrC* (A-C) and *rpoH* (D-F) was measured from the transcriptional and translational *lacZ* fusions and expressed as a corresponding β-galactosidase activity in Miller units as described in Methods. Data are represented by the mean (bar) + SEM (error bar) of n=4 biological samples in the bar graphs and as fold change in Miller units relative to the zero-hormone control in the line graphs. The experiments were performed at least thrice with reproducible results. Statistics: ANOVA test and a Dunnett's Multiple Comparison post-test (*, ** statistically different from 0 μM at $p<0.05$ and 0.01).

Figure S11

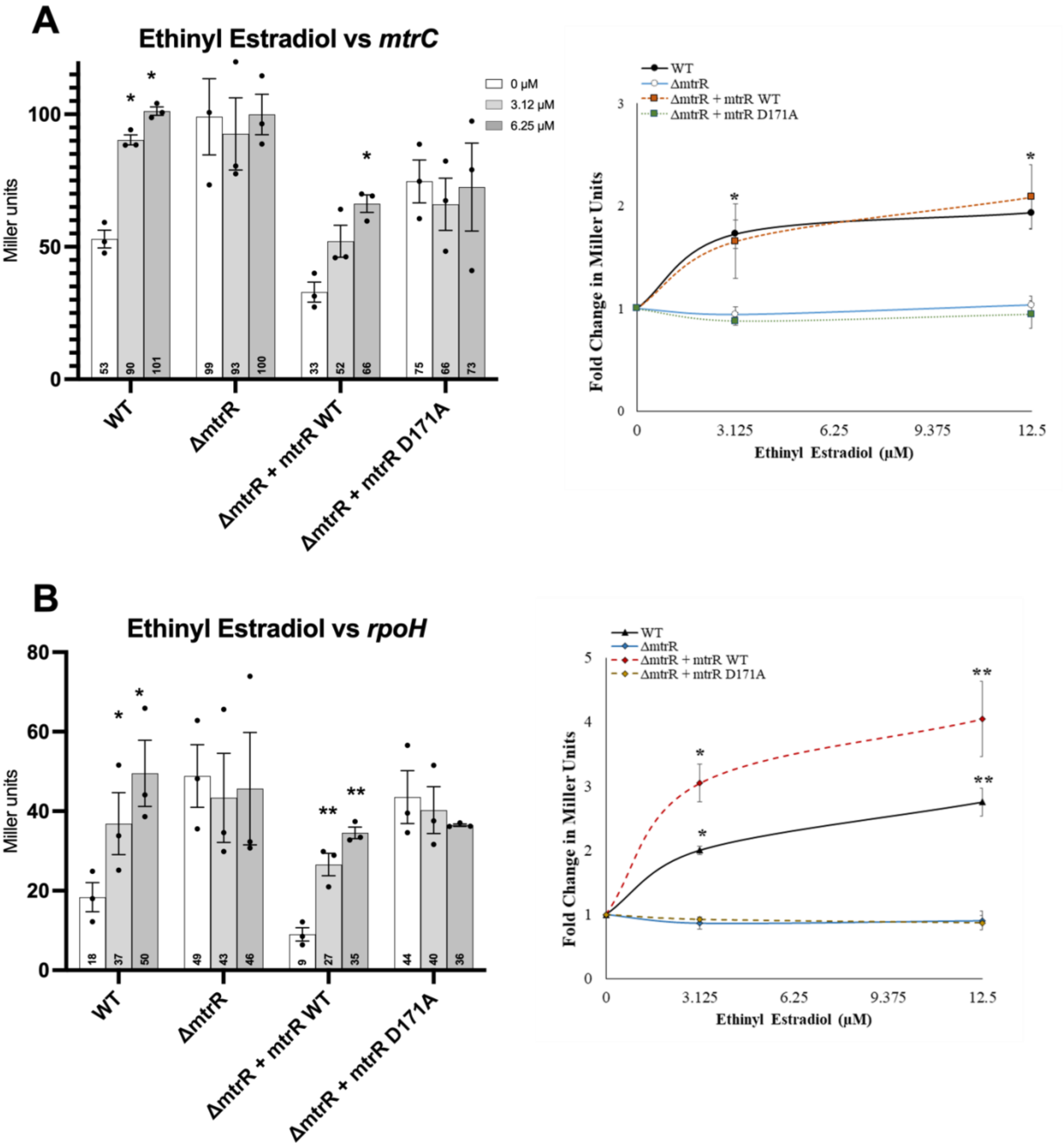

Fig S11. Ethinyl Estradiol induces the expression of gonococcal genes *mtrC* and *rpoH* in an MtrR-dependent manner. 10^8 CFU/mL of wild-type (WT) *mtrR* bearing strains (JC106 and JC108), *mtrR* deletion mutants (JC107 and JC109) and complemented strains in trans with WT *mtrR* (JC89 and JC91) and mutant allele *mtrR* D171A (JC101 and JC102) were grown in GC-broth to late exponential phase in increasing concentrations of ethinyl estradiol, statically in 96-well plates (37° C, 5% v/v CO_2). Expression of *mtrC* (A) and *rpoH* (B) was measured from the transcriptional and translational *lacZ* fusions and expressed as a corresponding β-galactosidase

activity in Miller units as described in Methods. Data are represented by the mean (bar) + SEM (error bar) of n=3 biological samples in the bar graphs and as fold change in Miller units relative to the zero-hormone control in the line graphs. The experiments were performed at least thrice with reproducible results. Statistics: ANOVA test and a Dunnett's Multiple Comparison post-test (*, ** statistically different from 0 μM at $p<0.05$ and 0.01)

Supplementary References

1 Sparling, P. F., Sarubbi, F. A., Jr. & Blackman, E. Inheritance of low-level resistance to penicillin, tetracycline, and chloramphenicol in Neisseria gonorrhoeae. *J Bacteriol* **124**, 740-749 (1975). https://doi.org:10.1128/jb.124.2.740-749.1975

2 Folster, J. P. & Shafer, W. M. Regulation of mtrF expression in Neisseria gonorrhoeae and its role in high-level antimicrobial resistance. *J Bacteriol* **187**, 3713-3720 (2005). https://doi.org:10.1128/jb.187.11.3713-3720.2005

3 Kandler, J. L. *et al.* The MisR Response Regulator Is Necessary for Intrinsic Cationic Antimicrobial Peptide and Aminoglycoside Resistance in Neisseria gonorrhoeae. *Antimicrob Agents Chemother* **60**, 4690-4700 (2016). https://doi.org:10.1128/aac.00823-16

4 Silver, L. E. & Clark, V. L. Construction of a translational lacZ fusion system to study gene regulation in Neisseria gonorrhoeae. *Gene* **166**, 101-104 (1995). https://doi.org:10.1016/0378-1119(95)00605-6

5 Mehr, I. J. & Seifert, H. S. Random shuttle mutagenesis: gonococcal mutants deficient in pilin antigenic variation. *Mol Microbiol* **23**, 1121-1131 (1997). https://doi.org:10.1046/j.1365-2958.1997.2971660.x

6 Beggs, G. A. *et al.* Structural, Biochemical, and In Vivo Characterization of MtrR-Mediated Resistance to Innate Antimicrobials by the Human Pathogen Neisseria gonorrhoeae. *J Bacteriol* **201** (2019). https://doi.org:10.1128/JB.00401-19

7 Beggs, G. A. *et al.* Structures of Neisseria gonorrhoeae MtrR-operator complexes reveal molecular mechanisms of DNA recognition and antibiotic resistance-conferring clinical mutations. *Nucleic Acids Res* **49**, 4155-4170 (2021). https://doi.org:10.1093/nar/gkab213
