# Supplementary material for: Hormonal steroids induce multidrug resistance and stress response genes in Neisseria gonorrhoeae by binding to MtrR
Source: Nat Commun. 2024 Feb 7;15:1153. doi: 10.1038/s41467-024-45195-1 (PMC10850145; doi:10.1038/s41467-024-45195-1)
Supplement: Supplementary file 3 — Description of Additional Supplementary Files [file 41467_2024_45195_MOESM3_ESM.pdf]

Description of Additional Supplementary Files:

Supplementary Movie 1: Induction movement of MtrR. Morph video of induced MtrR bound to β-estradiol and MtrR bound to the mtrCDE operator site. Key conformational changes occur in the HTH domain, α4, and the loop formed by residues 114-122, between helices α6 and α7, (opaque blue).
