# Supplementary material for: Hormonal steroids induce multidrug resistance and stress response genes in Neisseria gonorrhoeae by binding to MtrR
Source: Nat Commun. 2024 Feb 7;15:1153. doi: 10.1038/s41467-024-45195-1 (PMC10850145; doi:10.1038/s41467-024-45195-1)
Supplement: Supplementary file 5 — Reporting Summary [file 41467_2024_45195_MOESM5_ESM.pdf]

nature portfolio

Corresponding author(s): Richard G. Brennan

Last updated by author(s): December 19, 2023

Reporting Summary

Nature Portfolio wishes to improve the reproducibility of the work that we publish. This form provides structure for consistency and transparency in reporting. For further information on Nature Portfolio policies, see our Editorial Policies and the Editorial Policy Checklist.

Statistics

For all statistical analyses, confirm that the following items are present in the figure legend, table legend, main text, or Methods section.

n/a	Confirmed	
☐	☒	The exact sample size (n) for each experimental group/condition, given as a discrete number and unit of measurement
☐	☒	A statement on whether measurements were taken from distinct samples or whether the same sample was measured repeatedly
☐	☒	The statistical test(s) used AND whether they are one- or two-sided<br>*Only common tests should be described solely by name; describe more complex techniques in the Methods section.*
☐	☒	A description of all covariates tested
☐	☒	A description of any assumptions or corrections, such as tests of normality and adjustment for multiple comparisons
☐	☒	A full description of the statistical parameters including central tendency (e.g. means) or other basic estimates (e.g. regression coefficient) AND variation (e.g. standard deviation) or associated estimates of uncertainty (e.g. confidence intervals)
☐	☒	For null hypothesis testing, the test statistic (e.g. F, t, r) with confidence intervals, effect sizes, degrees of freedom and P value noted<br>*Give P values as exact values whenever suitable.*
☒	☐	For Bayesian analysis, information on the choice of priors and Markov chain Monte Carlo settings
☒	☐	For hierarchical and complex designs, identification of the appropriate level for tests and full reporting of outcomes
☒	☐	Estimates of effect sizes (e.g. Cohen's d, Pearson's r), indicating how they were calculated

Our web collection on statistics for biologists contains articles on many of the points above.

Software and code

Policy information about availability of computer code

Data collection	Advance Light Source (ALS) on beamline 5.0.2 and 5.0.1
Data analysis	Phenix 1.19, Coot 0.9.6, Pymol 2.5.4, ORIGIN 7.0 (Microcal Inc), Prism (Graphpad Software 9, 10), LigPlot+ v.2.2

For manuscripts utilizing custom algorithms or software that are central to the research but not yet described in published literature, software must be made available to editors and reviewers. We strongly encourage code deposition in a community repository (e.g. GitHub). See the Nature Portfolio guidelines for submitting code & software for further information.

Data

Policy information about availability of data

All manuscripts must include a data availability statement. This statement should provide the following information, where applicable:

- Accession codes, unique identifiers, or web links for publicly available datasets
- A description of any restrictions on data availability
- For clinical datasets or third party data, please ensure that the statement adheres to our policy

The coordinates and structure factors from this study have been deposited in the RCSB Protein Data Bank with the accession codes 8FW8, 8FW0, 8FW3, and 8SSH. Information on cell lines and plasmids will be available, after publication, upon reasonable request. Figure 1C contains structure of previously solved MtrR structure: PDB: 7JU3 [https://doi.org/10.2210/pdb7JU3/pdb]. Source data are provided with this paper.

Research involving human participants, their data, or biological material

Policy information about studies with human participants or human data. See also policy information about sex, gender (identity/presentation), and sexual orientation and race, ethnicity and racism.

Reporting on sex and gender	NA
Reporting on race, ethnicity, or other socially relevant groupings	NA
Population characteristics	NA
Recruitment	NA
Ethics oversight	NA

Note that full information on the approval of the study protocol must also be provided in the manuscript.

Field-specific reporting

Please select the one below that is the best fit for your research. If you are not sure, read the appropriate sections before making your selection.

[x] Life sciences [ ] Behavioural & social sciences [ ] Ecological, evolutionary & environmental sciences

For a reference copy of the document with all sections, see nature.com/documents/nr-reporting-summary-flat.pdf

Life sciences study design

All studies must disclose on these points even when the disclosure is negative.

Sample size	X-ray data were all collected to with 2-7 fold redundancy. For FP, ITC, MIC, and β-Galactosidase assays, no sample size calculations were performed. Sample size was determined to be adequate based on the consistency of measurable differences between groups. All experiments were done in triplicate. These sample sizes allowed for robust statistical analyses.
Data exclusions	No data were excluded from ITC, FP, β-Galactosidase, or MIC experiments and the crystallographic data were processed with XDS using the default parameters.
Replication	Crystallographic data were collected on one crystal per data set. Triplicates were performed for all FP and ITC assays. All attempts at replication for experiments were successful. X-ray structural coordinates were tested and validated by the MolProbity server before deposition to the Protein Data Bank and independent validation reports were then obtained from the Protein Data Bank upon data deposition.
Randomization	Crystallography samples were independent of each other. For ITC, FP, MIC, and β-Galactosidase assays all data were included in at least triplicate to ensure replication.
Blinding	Researchers were not blinded during crystallographic or other data collection or analysis. Prior Information regarding the samples is necessary to guide data collection and processing. For crystallographic analyses the identity of the sample (i.e. specific ligands) was necessary to enable model construction. For FP analyses the FP-ligand (DNA) and sample (protein) identities were necessary to enable proper experimental setup (such as the needed concentration of protein) and measurement range. For cellular experiments, the identify of the cells and treatments were necessary for experimental set up and data analysis.

Reporting for specific materials, systems and methods

We require information from authors about some types of materials, experimental systems and methods used in many studies. Here, indicate whether each material, system or method listed is relevant to your study. If you are not sure if a list item applies to your research, read the appropriate section before selecting a response.

Materials & experimental systems

n/a	Involved in the study
☒	☐ Antibodies
☒	☐ Eukaryotic cell lines
☒	☐ Palaeontology and archaeology
☒	☐ Animals and other organisms
☒	☐ Clinical data
☒	☐ Dual use research of concern
☒	☐ Plants

Methods

n/a	Involved in the study
☒	☐ ChIP-seq
☒	☐ Flow cytometry
☒	☐ MRI-based neuroimaging
